# Genome Mining and Metabolic Profiling Reveal Cytotoxic Cyclodipeptides in *Streptomyces hygrospinosus* var. Beijingensis

**DOI:** 10.3390/antibiotics11111463

**Published:** 2022-10-24

**Authors:** Dashan Zhang, Junbo Wang, Yongjian Qiao, Baixin Lin, Zixin Deng, Lingxin Kong, Delin You

**Affiliations:** State Key Laboratory of Microbial Metabolism, Joint International Research Laboratory of Metabolic and Developmental Sciences, School of Life Sciences and Biotechnology, Shanghai Jiao Tong University, Shanghai 200240, China

**Keywords:** genome mining, genetic dereplication, cyclodipeptide, OSMAC, cytotoxicity, *Streptomyces*

## Abstract

Two new cyclodipeptide (CDP) derivatives (**1–2**) and another seven known cyclodipeptides (**3–9**) were isolated from *Streptomyces* 26D9-414 by the genome mining approach combined with genetic dereplication and the “one strain many compounds” (OSMAC) strategy. The structures of the new CDPs were established on the basis of 1D- and 2D-NMR and comparative electronic circular dichroism (ECD) spectra analysis. The biosynthetic gene clusters (BGCs) for these CDPs were identified through antiSMASH analysis. The relevance between this *cdp* cluster and the identified nine CDPs was established by genetic interruption manipulation. The newly discovered natural compound **2** displayed comparable cytotoxicity against MDA-MB-231 and SW480 with that of cisplatin, a widely used chemotherapeutic agent for the treatment of various cancers.

## 1. Introduction

*Actinomyces* provide a rich source of natural products (NPs) with potential therapeutic applications, and modern “omics”-based technologies have revealed their potent potential for encoding diverse natural products [1]. Genome-guided discovery of clostrubin A [2], closthioamide [3] and cytotoxic benzolactones [4] has reinvigorated NP research, making it a more targeted and systematic research endeavor. To avoid the re-isolation of known NPs, the “genetic dereplication” strategy [5] and the “one strain many compounds” (OSMAC) approach [6] have been successfully used during large-scale culture for discovering NPs with novel skeletons (such as alterbrassinoids A-D [7] and waikikiamides [8]) and novel NPs derived from post-modifications (such as the branched cyclic peptide lyciumin [9] and highly modified polytheonamide-like peptides [10]).

Cyclodipeptides (CDPs), also called 2,5-diketopiperazines (DKPs), are the smallest cyclic peptides formed via the condensation of two α-amino acids. CDPs are mainly produced by *Streptomyces* [11]. CDPs exhibit important and diverse biological properties, such as antibacterial, antifungal, antiviral, antitumor, immunosuppressive and anti-inflammatory activities [12]. Owing to the great potential for activation of specific binding sites in enzymes or proteins, CDPs have become important pharmacophores in pharmaceutical chemistry [13]. Natural CDPs can be biosynthesized through two different machineries; one is catalyzed by the large multi-modular nonribosomal peptide synthetases (NRPSs), and the other is mediated by cyclodipeptide synthases (CDPSs) [14]. The former utilizes free amino acids, and the latter hijacks aminoacyl-tRNAs (AA-tRNAs) from primary metabolism [15]. Generally, CDPSs catalyze the production of representative 2,5-DKPs, which then will be modified by cyclodipeptide-tailoring enzymes (such as methyltransferases, prenyltransferases, oxidoreductases and cytochrome P450 enzymes) to form their intriguing molecular character [14,15].

Aiming at mining more natural products with structural diversity and bioactivity, the OSMAC strategy was carried out using *Streptomyces hygrospinosus* var. beijingensis, which is rich in secondary metabolites including tetramycin [16], anisomycin [17], nystatin and toyocamycin [18]. Among them, tetramycin, anisomycin and structurally related derivatives were high-yield products in the wild-type strain. To avoid the rediscovery of already characterized compounds and to reduce the interference effect of tetramycin and anisomycin, the BGCs of those compounds were genetically deleted and the resultant mutant strain (named *S. hygrospinosus* 26D9-414) was used as the starting strain in this study. When *S. hygrospinosus* 26D9-414 was incubated in a new medium different from the one used for tetramycin and anisomycin production, nine CDPs of two types, diketopiperazines with phenylalanine (**1** and **4–9**) and pyrazinones with arginine (**2** and **3**), were successfully identified. Among the identified CDPs, compounds **1** and **2** (argilein) were new compounds, and compound **4** was reported as a natural product for the first time here. Compound **4** has been used as an important substrate for antitumor spirotryprostatin B synthesis [19]. The other six CDPs have been reported before and were known as argvalin (**3**) [20], albonoursin (**5**) [21], 3,6-Dibenzylidene-2,5-dioxopiperazine (**6**) [22], 3-benzylidenepiperazine-2,5-dione (**7**) [23], 3-benzylidene-6-methylpiperazine-2,5-dione (**8**) [24] and 3-Benzyl-6-benzylidene-2,5-dioxopiperazine (**9**) [25], respectively. antiSMASH analysis of the genome sequence revealed a possible *cdp* cluster for the nine CDPs, and genetic deletion of *cdpA*-*C* confirmed the correlation of genes with compounds. Finally, the antibacterial and cytotoxic properties of **1–5** were evaluated.

## 2. Results and Discussion

**Figure 1:**
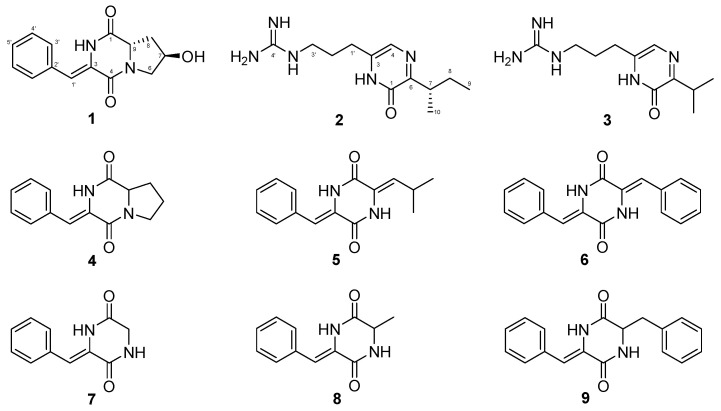
Structures of compounds **1–9**.

The “genetic dereplication” strain *Streptomyces* 26D9-414 [18], in which BGCs of tetramycin and anisomycin were deleted, was selected for OSMAC screening of new natural products. The original medium for anisomycin production and the other ten liquid media PYJ1-J10 were selected for the mining of new compounds. Comparative HPLC analysis of the secondary metabolites was conducted, and the metabolic profile of the PYJ1 medium gave many new peaks characteristic of absorption at 224 nm and 296 nm (Figure S1). Repeat rounds of fractionation alternating between silica gel chromatography and Sephadex LH-20 column chromatography followed by semi-preparative reversed-phase HPLC afforded compounds **1–9** (Figure 1). Their structures were elucidated by spectroscopic methods and HR-ESI-MS data.

**Figure 2:**
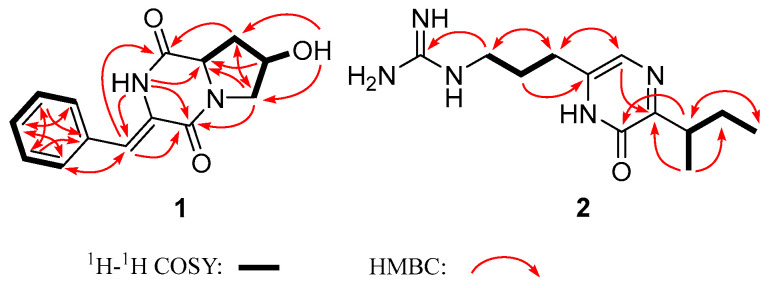
COSY and key HMBC correlations of compounds **1** and **2**.

Compound **1** was isolated as a white amorphous powder. The UV characteristic absorptions (224 nm and 296 nm) were similar to those of the already-known compound **4**. The similarity suggested a diketopiperazine moiety within **1**. Based on HR-ESI-MS ions at *m/z* 259.1079 [M + H]^+^, the molecular formula of **1** was determined as C_14_H_14_N_2_O_3_. According to the NMR data, it contained nine degrees of unsaturation. The diketopiperazine nature of **1** was confirmed through the ^1^H and ^13^C spectroscopic data analysis (Table 1), in which two amidic carbonyls (C-1 and C-4) were observed (Figure 1). The ^1^H NMR combined with H-H COSY spectrum showed characteristic 4-hydroxyproline residues at *δ_H_* 3.71 (1H, dd, *J* = 12.7, 4.7 Hz), 3.32 (1H, d, *J* = 12.7 Hz), 4.34 (1H, m), 2.12 (1H, dd, *J* = 12.6, 6.4 Hz), 2.01 (1H, td, *J* = 12.6, 4.4 Hz), 4.58 (1H, dd, *J* = 12.6, 6.4 Hz), 5.17 (1H, d, *J* = 2.9 Hz). Meanwhile, the ^13^C and 2D NMR spectrum of **1** revealed an α,β-unsaturated phenylalanine residue (*δ_C_* 158.7, 133.5, 129.3, 128.6, 127.9, 114.7) (Table 1). Finally, according to the HMBC correlations of H-2/C-9 and H-6/C-4, two fragments concatenated to form diketopiperazine (Figure 2). Therefore, the planar structure of compound **1** was a new pyrrolidine-containing and hydroxylated analog of compound **4**.

**Figure 3:**
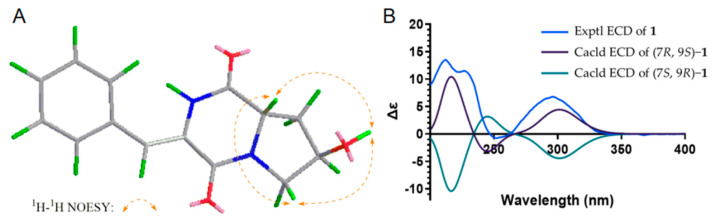
Key NOESY correlations and ECD curves of compound **1**.

The relative configuration of compound **1** was determined based on the NOESY spectrum (Figure 3A). The correlations between H-9 and 7-OH instead of H-9 and H-7 indicated that H-7 is located on the opposite side of H-9. Based on the NOE cross-peaks between H-2 and H-3′, the configurations of the β,γ-unsaturated bond in **1** were assigned as (*Z*). The absolute configuration was established by electronic circular dichroism (ECD), and the experimental ECD spectra matched well with the calculated ECD curves of 7*R*, 9*S* (Figure 3B). Considering the biosynthetic origins, the *S* configuration at C-9 was consistent with natural L-proline. Taken together, compound **1** was identified as (7*R*,9*S*)-3-((*Z*)-benzylidene)-7-hydroxy-hexahydropyrrolo [1,2-a] pyrazine-1,4-dione.

**Figure 4:**
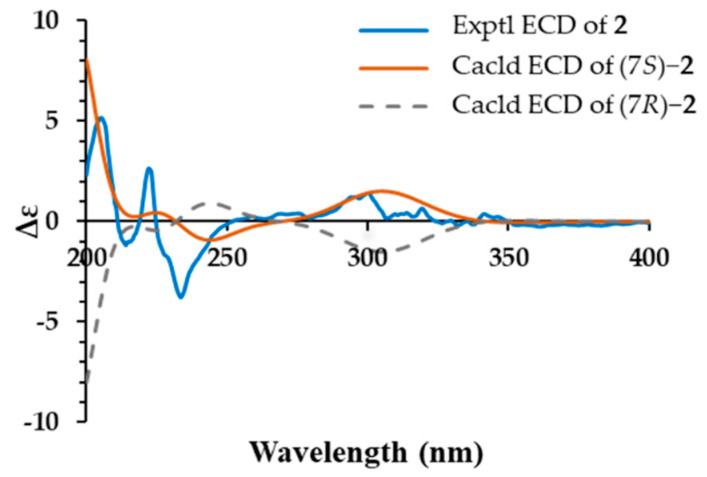
Experimental and calculated ECD curves of compound **2**.

Compound **2** was detected as an intracellular product with different UV spectra from compound **1**. The UV characteristic absorptions of **2** (228 nm and 322 nm) were more consistent with the known argvalin (**3**) [20]. Its molecular formula C_12_H_21_N_5_O was established on the basis of HR-ESI-MS data *m*/*z* 252.1823 [M + H]^+^ (Cal. 252.1819), which increased by 14 Da compared to **3**. The 1D NMR data of **2** were similar to those of **3** except for the two methyl groups with different chemical shifts and the presence of an extra methylene at δ_C_ 27.1 (Table 1). These data suggest that isoleucine, rather than valine, was condensed with arginine, leading to the formation of compound **2** as a new pyrazine derivative. Considering the arginine origin, compound **2** was named argilein. This is the third arginine-containing pyrazine derivative found in natural products. Similarly, the absolute configuration of **2** was 7*S*, which was consistent with the L-isoleucine (Figure 4).

The compounds **1–5** were evaluated for cytotoxicity against human lung carcinoma (A549), human leukemia (HL60), human hepatocellular carcinoma (SMMC-7721), human colon cancer (SW480) and human breast carcinoma (MDA-MB-231) cell lines by MTS assay. Among the five compounds, only compound **2** exhibited selective inhibitory activity against MDA-MB-231 and SW480, the IC_50_ values of which were 18.26 μM and 13.42 μM, respectively (Table S3). The cytotoxicity of compound **2** was comparable with that of cisplatin, which has been widely used as a chemotherapeutic agent for the treatment of various cancers [26]. As to the antibiotic activity, all compounds showed no obvious inhibitory activity against all tested bacteria and fungi, except for compound **2**, which exhibited weak activity against *Xanthomonas albilineans, Candida albicans* and *Candida sake* with MIC values of 0.25 mg/mL, 1.0 mg/mL and 1.0 mg/mL, respectively (Table S4). This finding was consistent with the reported weak antibacterial activities of CDPs (MICs of 0.5–10 mg/mL) [27,28].

**Figure 5:**
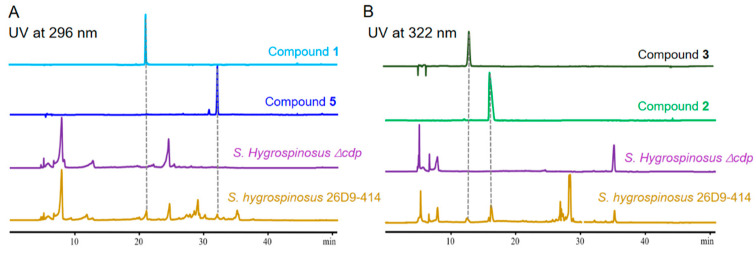
HPLC analysis of metabolites in *S. hygrospinosus* 26D9-414 and in mutant *S. hygrospinosus* Δ*alb.* (**A**) The UV detection of piperazines were performed at 296 nm; (**B**) The UV detection of pyrazinones at 322 nm.

To correlate BGCs with the isolated nine diketopiperazines, antiSMASH analysis of the genome sequence of *S. hygrospinosus* var. beijingensis was conducted. The arrangement and sequence of genes within the *cdp* cluster showed high similarity with those of the *alb* cluster, which was reported to be responsible for albonoursin (**5**) production (Figure S2) [21]. *albC* encodes cyclodipeptide synthase (CDPS), which catalyzes the cyclic dipeptide precursor formation. The heterologous expression of *albC* led to the synthesis of various cyclodipeptides, including cyclo(Phe-Pro) [29,30], the possible precursor of compounds **1** and **4**. The deletion of *cdpA-cdpC* abolished the production of nine cyclodipeptides (Figure 5 and Figure S4). To the best of our knowledge, this is the first finding of a cyclodipeptide synthesized by CDPS using arginine and also the first report of a proline-derived cyclodipeptide (compounds **1** and **4**) from the original producing strain.

Cyclodipeptide oxidases (CDOs) AlbA and AlbB usually catalyze the dehydrogenation of cyclodipeptides to form dehydrogenated cyclodipeptide derivatives [31]. Whether the hydroxyl group in **1** and pyrazinone in **2** are catalyzed by CDO candidates CdpA and CdpB still awaits discovery. CDPSs and CDOs both possess broad substrate selectivity and can be used to synthesize various dehydrogenated cyclodipeptide derivatives, which serve as important precursors for the development of pharmaceutical intermediates [30,32]. Gene c-blast analysis revealed that *cdp* gene analogs were mainly distributed in *Streptomyces* and *Nocardiopsis*, and a few were also found in *Nonomuraea*, *Goodfellowiella*, *Bailinhaonella*, *Saccharopolyspora* and *Actinomadura* (Figure S5).

## 3. Conclusions

Modern “omics”-based technologies have revealed the potent potential of *Actinobacteria* for encoding natural products with diverse structures and biologically active compounds. To reveal the diversity of NPs encoded by *Streptomyces hygrospinosus* var. beijingensis, the “genetic dereplication” strategy and OSMAC approach were used in this study. Nine CDP derivatives of two types were identified from *S. hygrospinosus* 26D9-414 through the genome mining strategy. The relevance between the *cdp* cluster and all the isolated CDPs was confirmed by genetic manipulation. These findings increase the repertoire of natural DKPs and reveal a CDPS with a broad range of substrates that could be developed as a biocatalyst for the future development of therapeutic agents.

## 4. Material and Methods

### 4.1. General Experimental Procedures

Optical rotations were recorded with a JASCO P-2000 digital polarimeter. UV spectra were recorded on a Thermofisher Evolution 300 UV-vis spectrophotometer. The 1D-NMR and 2D-NMR spectra were obtained on a Bruker AVANCE III 600 MHz spectrometer with TMS as an internal standard. HR-ESI-MS spectra were recorded on an Agilent 1290 HPLC system coupled to a 6230 TOF system mass spectrometer. ECD spectra were recorded using a JASCO J-1500-150ST. HPLC analysis and semi-preparative HPLC were performed with Agilent 1260 HPLC system using an Agilent ZORBAX SB-C18 column (5 μm, 4.6 × 250 mm) and an Agilent ZORBAX SB-C18 column (5 μm, 9.4 × 250 mm), respectively. All comparative studies of crude extracts obtained based on the OSMAC strategy were based on HPLC analysis, the mobile phases were CH_3_OH-H_2_O and 1‰ formic acid or trifluoroacetic acid in the water. The gradient was chosen as CH_3_OH-H_2_O: 5% 0–5 min, 5–50% 5–30 min, 50–95% 30–45 min, 95% 45–50 min, 95–5% 50–51 min, 5% 51–60 min, 0.5 mL/min. The HPLC methods used for the separation of compounds **1–9** are described in detail in Section 4.5. Silica gel (100–200, 200–300 mesh, Qingdao Haiyang Chemical Co., Ltd., Qingdao, China) and Sephadex LH-20 gel (Uppsala, Sweden) were used for column chromatography (CC). Precoated silica gel GF254 plates (Qingdao Marine Chemical Ltd., Qingdao, China) were used for TLC monitoring combined with UV light and 10% H_2_SO_4_ in EtOH. Taq DNA polymerase and KOD-plus high-fidelity polymerase were obtained from Takara. All restriction enzymes were purchased from Thermo Scientific or Vazyme Biotech Co., Ltd. E.Z.N.A. Gel Extraction Kit and Plasmid Mini Kit were purchased from OMEGA. PCR primers were synthesized by GENEWIZ. All solvents used for CC were of analytical grade (Shanghai Chemical Reagents Co., Ltd., Shanghai, China), and solvents used for HPLC were of HPLC grade (Sigma-Aldrich, St. Louis, MO, USA).

### 4.2. Bacterial Strains, Plasmids, Primers and Culture Conditions

The strains, plasmids and primers used in this study were listed in Tables S1 and S2. *Streptomyces* and its derivatives were grown at 30 °C on solid SFM medium (2% mannitol, 2% soya flour and 1.5% agar) for sporulation and conjugation, and in TSBY liquid medium (3% tryptone soy broth, 10.3% sucrose and 0.5% yeast extract) for the isolation of chromosomal DNA [33]. All *E. coli* strains including DH10B and ET12567/pUZ8002 were grown in liquid Luria–Bertani (LB) medium or on LB agar at 37 °C. Apramycin (50 μg/mL) and trimethoprim (50 μg/mL) were used when necessary. All plasmid subcloning experiments were performed in *E. coli* DH10B following standard protocols. General procedures for *E. coli* or *Streptomyces* manipulation were carried out according to the published procedures [34].

### 4.3. Construction of S. hygrospinosus Δcdp Mutant

To construct the *cdpA-cdpC* deletion mutant, the 1624 bp DNA fragment covering total *cdpA-cdpC* was substituted by *aac(3)IV* + *oriT* cassette (Apr^R^ gene), amplified from pIJ773 primers cdp-apr-P1/P2 and cdp-apr-P1/P2 (Table S2). Two homologous arms of 2004 bp and 1964 bp containing the upstream and downstream regions flanking *cdpA-cdpC* were amplified by PCR with primers cdp-L-P1/P2 and cdp-R-P1/P2, respectively (Table S2). The entire PCR product was cloned into the *BamHI/EcoRI-*digested pJTU1278, generating the recombinant plasmid vector pZDS-1*,* using the Vazyme one-step cloning kit (Vazyme Biotech Co., Ltd., Nanjing, China). The resultant plasmid was firstly transferred into *E. coli* ET12567/pUZ8002 and then introduced into *S. hygrospinosus* 26D9-414 strain for the construction of *cdpA-C*-deleted strain *S. hygrospinosus* Δ*cdp*. According to the previously described procedure [35], the double-crossover strains were obtained through antibiotic selection and confirmed by PCR verification using primers cdp1-P1/P2 and apr-P1/P2 (Table S2).

### 4.4. Strain Fermentation and Chemical Analysis

The mutant *S. hygrospinosus* 26D9-414 was cultivated in TSBY liquid medium at 30 °C for 2 days to afford seed broth. The seed broth was next inoculated into a fermentation medium (5% (*v*/*v*)) and incubated at 30 °C with shaking for a further 6 days. Ten different fermentation liquid media (Table S4) including the original medium (containing 1% corn starch, 2% soluble starch, 1% soya flour, 0.02% KH_2_PO_4_, 0.3% NaCl, 0.3% NH_4_Cl and 0.4% CaCO_3_ per liter) were selected for tetramycin and anisomycin production [36]. The EtOAc extracts of all fermentation broths were analyzed by high-performance liquid chromatography (HPLC). For the accumulation of nine CDPs, medium PYJ1 (containing 3% soluble starch, 4% glucose, 1% glycerin, 1.5% tryptone soy broth, 1% beef extract, 1% peptone, 0.65% yeast extract, 0.05% MgSO_4_, 0.1% NaCl and 0.2% CaCO_3_ per liter) was used.

### 4.5. Fermentation and Isolation

Large-scale fermentation for the isolation and purification of DKPs was conducted according to the standard method described before. A total of 10 L fermentation broth was centrifuged to afford the mycelia and the liquid phase. The liquid phase was extracted with an equal volume of EtOAc three times at room temperature. The EtOAc crude extract was concentrated under reduced pressure to yield dark brown matter (2.6 g). The crude extract was separated using a column of silica gel eluted with CHCl_3_/MeOH (from 100:1 to 1:1, *v*/*v*) to obtain eight fractions (Fr.A-Fr.H). Then Fr.B was directly separated by semi-preparative HPLC (CH_3_OH-H_2_O: 10% 0–5 min, 10–90% 5–40 min, 1.5 mL/min, 296 nm) to afford compounds **1** (22.4 mg, *t_R_* = 23 min) and **7** (16.8 mg, *t_R_* = 22 min); Fr.C was subjected to Sephadex LH-20 column chromatography and elution with CHCl_3_/MeOH (1:1, *v*/*v*) to give four fractions (Fr.C1-C4); Fr.C3 was separated by semi-preparative HPLC (CH_3_OH-H_2_O: 5% 0–5 min, 5–80% 5–40 min, 1.5 mL/min, 296 nm) to afford compounds **4** (16.6 mg, *t_R_* = 27 min), **5** (6.3 mg, *t_R_* = 34 min), **6** (2.6 mg, *t_R_* = 36 min), **8** (12.1 mg, *t_R_* = 26 min) and **9** (3.2 mg, *t_R_* = 30 min). The mycelia were extracted with acetone (1 L) and ultrasound for 2 h, and the organic solvents were dried under vacuum to yield a dark brown crude extract (1.1 g). The extract was separated into six fractions (Fr.a-Fr.f) by silica gel column chromatography using CHCl_3_/MeOH mixtures of increasing polarities (100:1 to 5:1, *v*/*v*). Fr.e was separated by semi-preparative HPLC (CH_3_OH-H_2_O: 5–50% 30 min, 1.5 mL/min, 322 nm, 1‰ TFA in water) to afford compounds **2** (7.4 mg, *t_R_* = 22 min) and **3** (5.5 mg, *t_R_* = 20 min).

**Compound 1:** white powder; [α${]}_{D}^{25}$ +13.5 (c 0.2 MeOH); UV (MeOH) *λ*_max_ (log *ε*) 214 (3.31), 299 (3.32) nm; ^1^H NMR (600 MHz, DMSO-*d_6_*) and ^13^C NMR (150 MHz, DMSO-*d_6_*) data in Table 1; HR-ESI-MS *m/z* 259.1079 [M + H]^+^ (calcd for C_14_H_15_N_2_O_3_^+^, 259.1077).

**Compound 2:** pale-yellow powder; [α${]}_{D}^{25}$ −12.1 (c 0.2 MeOH); UV (MeOH) *λ*_max_ (log *ε*) 230 (3.12), 323 (3.17) nm; ^1^H NMR (600 MHz, DMSO-*d_6_*) and ^13^C NMR (150 MHz, DMSO-*d_6_*) data in Table 1; HR-ESI-MS *m/z* 252.1823 [M + H]^+^ (calcd for C_12_H_22_N_5_O^+^, 252.1819).

### 4.6. ECD Calculations

Conformational analyses for compounds **1–2** were performed via Spartan’14 software using the MMFF94 molecular mechanics force field calculation. Conformers within a 10 kcal/mol energy window were generated and optimized using DFT calculations at the B3LYP/6-31G(d) level. Conformers with a Boltzmann distribution over 1% were chosen for the ECD calculations in MeOH at the B3LYP/6-311 + G (2d, p) level. The IEF-PCM solvent model for MeOH was used. The calculated ECD spectra were obtained by DFT and time-dependent DFT (TD-DFT) using Gaussian 09 and analyzed using SpecDis v1.71.

### 4.7. Cytotoxicity Assays

To determine the cytotoxicity of compounds **1–5**, five human cancer cell lines (HL60, A549, SMMC-7721, SW480, MDA-MB-23) were evaluated by MTS assay. Each cell line was exposed to the tested compounds at concentrations of 40, 8, 1.6, 0.32 and 0.064 μM in triplicate. Cell viability was determined using MTS Kit according to the manufacturer’s instructions [37].

## Figures and Tables

**Table 1:** ^1^H (600 MHz) and ^13^C (150 MHz) NMR data for **1** and **2**.

No.	1		2	
	*δ_H_*, Mult (*J* in Hz)	*δ_C_*, Type	*δ_H_*, Mult (*J* in Hz)	*δ_C_*, Type
1		167.5, C		158.8, C
2	10.03, s		12.10, s	
3		128.6, C		137.5, C
4		158.7, C	7.84, s	120.1, C
5				
6	3.71, dd (12.7, 4.7)	54.5, CH_2_		156.0, C
	3.32, d (12.7)			
7	4.34, m	66.6, CH	3.08, m	35.7, CH
8	2.12, dd (12.6, 6.4)	37.3, CH_2_	1.68, m	27.1, CH_2_
	2.01, td (12.6, 4.4)		1.41, m	
9	4.58, dd (12.6, 6.4)	56.8, CH	0.79, t (7.4)	11.9, CH_3_
10			1.07, d (6.9)	17.8, CH_3_
1′	6.67, s	114.8, CH	2.43, t (7.5)	26.7, CH_2_
2′		133.6, C	1.78, m	27.2, CH_2_
3′	7.54, d (7.6)	129.4, CH	3.10, t (6.4)	40.1, CH_2_
4′	7.40, t (7.6)	128.6, CH		156.9, C
5′	7.30, t (7.6)	128.0, CH		
7-OH	5.17, d (2.9)			

See Supplementary Materials for NMR spectra. Spectra were recorded in DMSO*-d_6_*.

## Data Availability

All data generated or analyzed during this study are included in this published article.

## Supplementary Materials

The following supporting information is free of charge and can be downloaded at: https://www.mdpi.com/article/10.3390/antibiotics11111463/s1. Figure S1: HPLC analysis of fermentation products of *S*. *hygrospinosus* 26D9-414 from eleven different mediums. Figure S2: Biosynthetic analysis of CDPs. Figure S3: Schematic construction and PCR verification of S. hygrospinosus Δ*cdp* mutant. Figure S4: HPLC analysis of metabolites in *S*. *hygrospinosus* 26D9-414 and in *S*. *hygrospinosus* Δ*cdp* mutant. Figure S5: Distribution statistics of putative *cdp* gene analogues. Figures S6–S12: 1D and 2D NMR, HR-ESI-MS, and UV spectra of compound **1**. Figures S13–S20: 1D and 2D NMR, HR-ESI-MS, and UV spectra of compound **2**. Table S1: Strains and plasmids used in this study. Table S2: Primers used in this study. Table S3: Cytotoxicity assay of compounds **1–5**. Table S4: Antibacteria activity assay of compounds **1** and **2**. Table S5: Ten mediums in this study. References [18,21,38,39,40,41,42,43,44,45,46] are cited in Supplementary Materials file.
