# Supplementary material for: Genome Mining and Metabolic Profiling Reveal Cytotoxic Cyclodipeptides in Streptomyces hygrospinosus var. Beijingensis
Source: Antibiotics (Basel). 2022 Oct 24;11(11):1463. doi: 10.3390/antibiotics11111463 (PMC9686873; doi:10.3390/antibiotics11111463)
Supplement: Supplementary file 1 [file antibiotics-11-01463-s001.zip › antibiotics-1996958-supplementary.pdf]

**Supplementary Materials**

# Genome mining and metabolic profiling reveal cytotoxic cyclodipeptides in *Streptomyces hygrospinosus* var. beijingensis

Dashan Zhang†, Junbo Wang†, Yongjian Qiao†, Baixin Lin†, Zixin Deng†, Lingxin Kong†*, Delin You†*

† State Key Laboratory of Microbial Metabolism, Joint International Research Laboratory of Metabolic and Developmental Sciences, School of Life Sciences and Biotechnology, Shanghai Jiao Tong University, Shanghai 200240, China;

*Corresponding author E-mail: konglingxin7@sjtu.edu.cn; dlyou@sjtu.edu.cn.

## Table of Contents


| **Contents** | **pages** |
|---|---|
| ***Table S1.*** Strains and plasmids used in this study | 2 |
| ***Table S2.*** Primers used in this study | 2 |
| ***Table S3.*** Cytotoxicity assay of compounds **1-5** | 2 |
| **Table S4.** Antibacteria activity assay of compounds **1** and **2**. | 3 |
| ***Table S5.*** Ten mediums in this study. | 3 |
| ***Figure S1.*** HPLC analysis of fermentation products of *S. hygrospinosus* 26D9-414 from eleven different mediums. | 4 |
| ***Figure S2.*** Biosynthetic analysis of CDPs. | 5 |
| ***Figure S3.*** Schematic construction and PCR verification of *S. hygrospinosus* ⊿*cdp* mutant. | 6 |
| ***Figure S4.*** HPLC analysis of metabolites in *S. hygrospinosus* 26D9-414 and in *S. hygrospinosus Δcdp* mutant. | 7 |
| ***Figure S5.*** Distribution statistics of putative *cdp* gene analogues. | 8 |
| ***Figure S6-12.*** 1D and 2D NMR, HR-ESI-MS, and UV spectra of compound **1** | 9-12 |
| ***Figure S13-20.*** 1D and 2D NMR, HR-ESI-MS, and UV spectra of compound **2** | 13-16 |

***Table S1.*** Strains and plasmids used in this study

| Strain or plasmid | Description | Source or reference |
|---|---|---|
| ***Streptomyces* strains** | | |
| *S. hygrospinosus* 26D9-414 | *ani* and *tetr* deletion mutant | [18] |
| *S. hygrospinosus Δcdp* | *Ani, tetr* and *cdp* deletion mutant | This study |
| ***E. coli* strains** | | |
| DH10B | General molecule cloning strain | Gibco BRL |
| ET12567/pUZ8002 | Intergeneric conjugation strain | [38] |
| **Plasmids** | | |
| pJTU1278 | template plasmid for gene deletion | [39] |
| pIJ773 | template for PCR amplification of *Apr* gene | [40] |
| pZDS-1 | constructed plasmid for the *cdp* genes deletion | This study |

***Table S2.*** Primers used in this study

| Primer | Sequence (5'–3') | Use(s) |
|---|---|---|
| cdp-L-P1 | GGCCGCTCTAGAACTAGTGGATCCTGATGTTGGCGGCGAAGATGAC | Amplification of *cdp* fragment for insertion into pJTU1278 by one-step cloning. |
| cdp-L-P2 | GAAGCAGCTCCAGCCTACACGATTGACCTGCGGCTGGATTC | |
| cdp-apr-P1 | GAATCCAGCCGCAGGTCAATCGTGTAGGCTGGAGCTGCTTC | Amplification of *Apr* cassette from pIJ773 |
| cdp-apr-P2 | TGTTGTGGCACCGCGACAAATTCCGGGGATCCGTCGACC | |
| cdp-R-P1 | GGTCGACGGATCCCCGGAATTTGTCGCGGTGCCACAACA | Amplification of *cdp* fragment for insertion into pJTU1278 by one-step cloning. |
| cdp-R-P2 | GTATCGATAAGCTTGATATCGAATTCAGCAGAAGATGAGCCCGAGTTC | |
| apr-P1 | CAGCGTCGTGTTGGCATCGT | Verification of *Δcdp* mutant strain |
| apr-P2 | ATCAGCGGTGGAGTGCAATGTC | |
| cdp1-P1 | GGTCCGAGATCGTCAAGAGCAT | Verification of *Δcdp* mutant strain |
| cdp1-P2 | GCAGCAGCATGGTGAGGTTCT | |

***Table S3.*** Cytotoxicity assay of compounds **1-5** ($IC_{50}$, μM)

| Compound | A549 | HL60 | SMMC-7721 | SW480 | MDA-MB-231 |
|---|---|---|---|---|---|
| **1** | >40 | >40 | >40 | >40 | >40 |
| **2** | >40 | >40 | >40 | **13.42 ± 0.78** | **18.26 ± 1.56** |
| **3** | >40 | >40 | >40 | >40 | >40 |
| **4** | >40 | >40 | >40 | >40 | >40 |
| **5** | >40 | >40 | >40 | >40 | >40 |
| **cisplatin** | - | - | - | 14.99 ± 0.09 | 14.03 ± 0.75 |

[a]Human lung carcinoma (A549), human leukemia cell (HL60), human hepatocellular carcinoma (SMMC-7721), human colon cancer cell (SW480) and human breast carcinoma

(MDA-MB-231 were tested for cytotoxicity of CDPs and cisplatin was used as positive control.

***Table S4***. Antibacterial activity assay of compounds **1-9**.

| Compound | *E. coli* | *P. aeruginosa* | *S. aureus* | *B. subtilis* | *X.albilineans* | *C. albicans* | *C. sake* |
|---|---|---|---|---|---|---|---|
| **1** | >1.0 | >1.0 | >1.0 | >1.0 | >1.0 | >1.0 | >1.0 |
| **2** | >1.0 | >1.0 | >1.0 | >1.0 | **0.25** | **1.0** | **1.0** |
| **3** | >1.0 | >1.0 | >1.0 | >1.0 | >1.0 | >1.0 | >1.0 |
| **4** | >1.0 | >1.0 | >1.0 | >1.0 | >1.0 | >1.0 | >1.0 |
| **5** | >1.0 | >1.0 | >1.0 | >1.0 | >1.0 | >1.0 | >1.0 |
| **6** | >1.0 | >1.0 | >1.0 | >1.0 | >1.0 | >1.0 | >1.0 |
| **7** | >1.0 | >1.0 | >1.0 | >1.0 | >1.0 | >1.0 | >1.0 |
| **8** | >1.0 | >1.0 | >1.0 | >1.0 | >1.0 | >1.0 | >1.0 |
| **9** | >1.0 | >1.0 | >1.0 | >1.0 | >1.0 | >1.0 | >1.0 |
| **apramycin** | 0.025 | 0.025 | 0.013 | 0.006 | 0.006 | - | - |
| **nystatin** | - | - | - | - | - | 0.004 | 0.002 |

Apramycin and nystatin as positive control.

***Table S5.*** Ten selected mediums in this study.

| Name | Composition |
|---|---|
| PYJ1 | Glucose 40 g, glycerol 10 g, soluble starch 30 g, soytone 15 g, beef extract 10 g, peptone 6.5 g, yeast extract 0.5 g, $MgSO_4$ 1 g, NaCl 2g, $CaCO_3$ 2,4 g in 1.0 L of distilled $H_2O$. [41] |
| PYJ2 | Sucrose 100 g, glucose 10 g, casamino acids 0.1 g, yeast extract 5 g, MOPS 21 g, $K_2SO_4$ 0.25 g, $MgCl_2{\cdot}6H_2O$ 10 g, $FeSO_4{\cdot}7H_2O$ 0.001 g, $MnCl_2{\cdot}7H_2O$ 0.001 g, $ZnSO_4{\cdot}7H_2O$ 0.001 g in 1.0 L of distilled $H_2O$, pH 7.0. [42] |
| PYJ3 | Soluble starch 15g, soy peptone 15 g, soybean meal 5 g, glycerol 15 g, $CaCO_3$ 2 g, sea salt 30 g in 1.0 L of distilled $H_2O$. [43] |
| PYJ4 | Glycerol 10 g, peptone 8.6 g, soybean meal 40 g, cottonseed meal 20 g, mannitol 97.3 g, ornithine 5.3 g, proline 5 g, $CaCl_2$ 5 g, $K_2HPO_4$ 8.4 g, $MgSO_4{\cdot}7H_2O$ 5g, $CuSO_4{\cdot}5H_2O$ 0.6 g, $MnSO_4{\cdot}H_2O$ 0.1 g, $FeSO_4{\cdot}7H_2O$ 0.05 g in 1.0 L of distilled $H_2O$. [44] |
| PYJ5 | Corn starch 24 g, glucose 10 g, peptone 3 g, beef extract 3 g, yeast extract 5 g, $CaCO_3$ 4 g in 1.0 L of distilled $H_2O$, pH 7.0. [45] |
| PYJ6 | glucose 10 g, Dextrin 25 g, oat meal 20 g, cottonseed meal 10 g, fish meal 5 g, yeast extract 2 g, $CaCO_3$ 3 g in 1.0 L of distilled $H_2O$, pH 7.2. [46] |
| PYJ7 | Soluble starch 30g, glucose 15 g，glycerol 5 g, $NaNO_3$ 5 g, $NH_4Cl$ 5 g, $K_2HPO_4$ 1 g, $KH_2PO_4$ 2 g, $MnSO_4{\cdot}7H_2O$ 2 g, $CaCO_3$ 2 g in 1.0 L of distilled $H_2O$. (This research) |
| PYJ8 | Dextrin 40 g, molasses 5 g, glucose 10 g，casamino acid 10 g，$MgSO_4{\cdot}7H_2O$ 1 g，$CaCO_3$ 2 g in 1.0 L of distilled $H_2O$, pH 7.5. (This research) |
| PYJ9 | Soluble starch 10 g, peptone 10 g, glycerol 10 g, mannitol 5 g, $(NH_4)_2HPO_4$ 1 g in 1.0 L of distilled $H_2O$, pH 7.0. (This research) |
| PYJ10 | glucose 25 g, soya flour 18.8 g, molasses 3.75 g, NZ Amine type A 1.25 g, $CH_3COONa$ 8 g, $CaCO_3$ 3 g in 1.0 L of distilled $H_2O$, pH 7.0. (This research) |

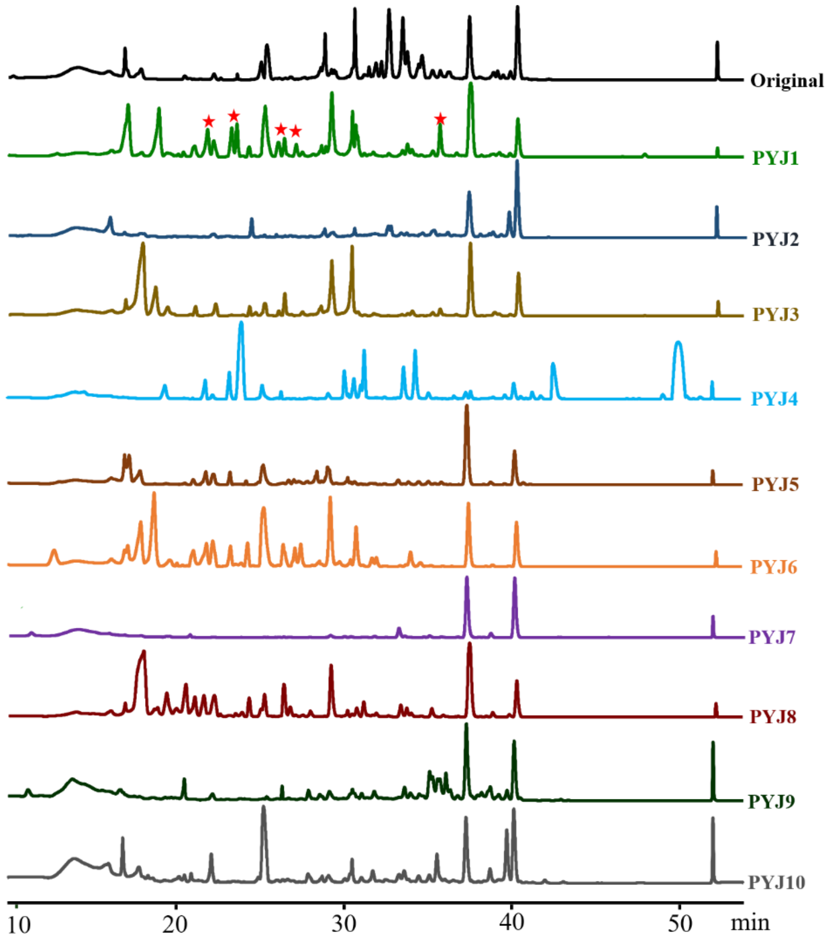


***Figure S1***. HPLC analysis of fermentation products of *S. hygrospinosus* 26D9-414 from eleven different mediums.

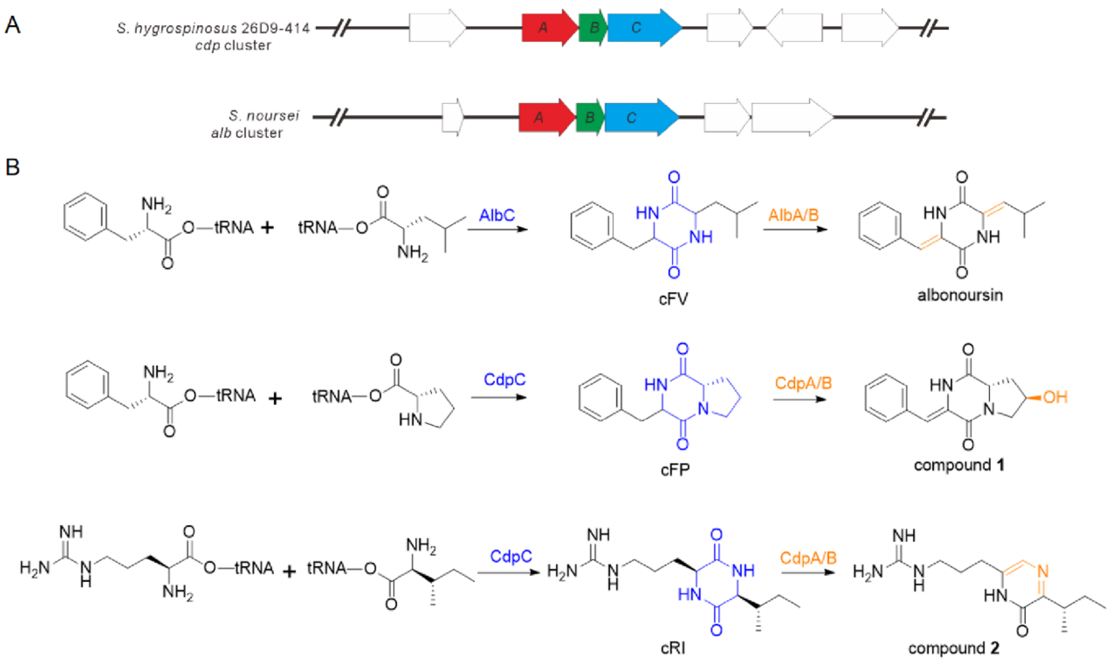


***Figure S2.*** Biosynthetic analysis of CDPs. (A) Comparative analysis of *cdp* cluster with the reported *alb* cluster. Proteins encoded by *alb* cluster for albonoursin biosynthesis: AlbA, cyclic dipeptide synthase (CDPS), AlbB and AlbC, cyclic dipeptide oxidase (CDO) [21]. (B) biosynthetic machineries for CDPs (compounds **1** and **2**) in this study.

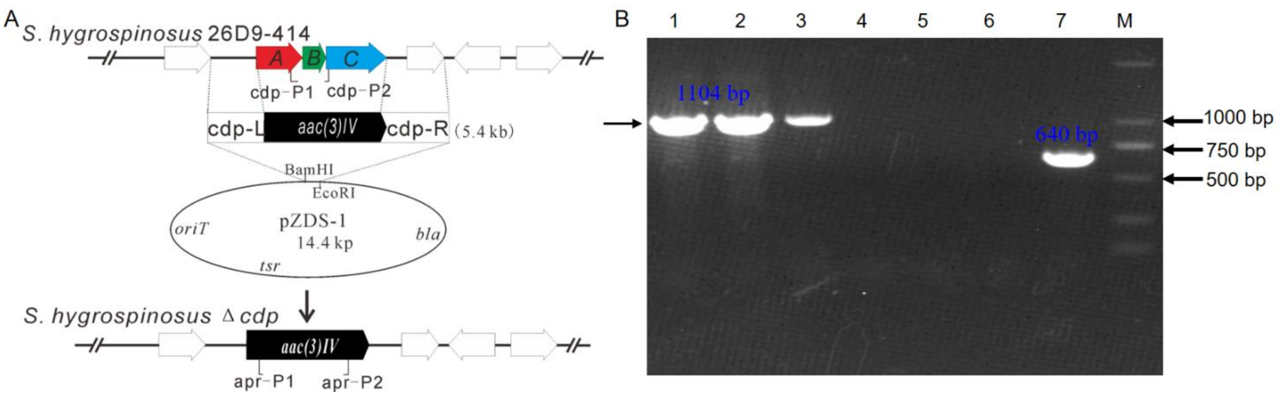


***Figure S3***. Schematic construction and verification of *S. hygrospinosus Δcdp* mutant. (**A**) Schematic representation of *cdpA-C* fragment replaced by *aac(3)IV* apramycin resistence gene, and then the mutated shuttle plasmid pZDS-1 was introduced into *S. hygrospinosus* 26D9-414 by intergeneric conjugation. (**B**) PCR verification of *S. hygrospinosus Δcdp* mutants using primers apr-L/R and cdp-L/R. Lane 1 and 2, genonme of two clone of *S. hygrospinosus Δcdp* mutant were used as template using primers apr-L/R; lane 3, pIJ773 was used as template for positive control; lane 4, genome of *S. hygrospinosus* 26D9-414 used as template for negative control; lane 5 and 6, genonme of two clone of *S. hygrospinosus Δcdp* mutant were used as template using primers cdp-L/R; lane 7, genome of *S. hygrospinosus* 26D9-414 used as template for negative control; M, *LD* DS™ 2000 kb DNA ladder.

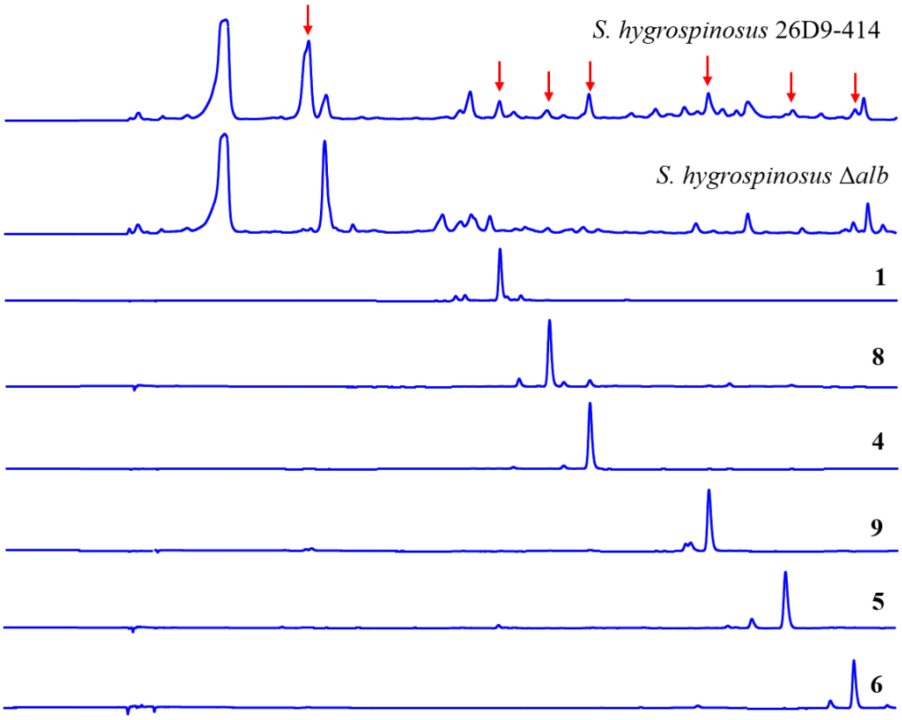


***Figure S4***. HPLC analysis of metabolites in *S. hygrospinosus* 26D9-414 and in *S. hygrospinosus Δcdp* mutant.

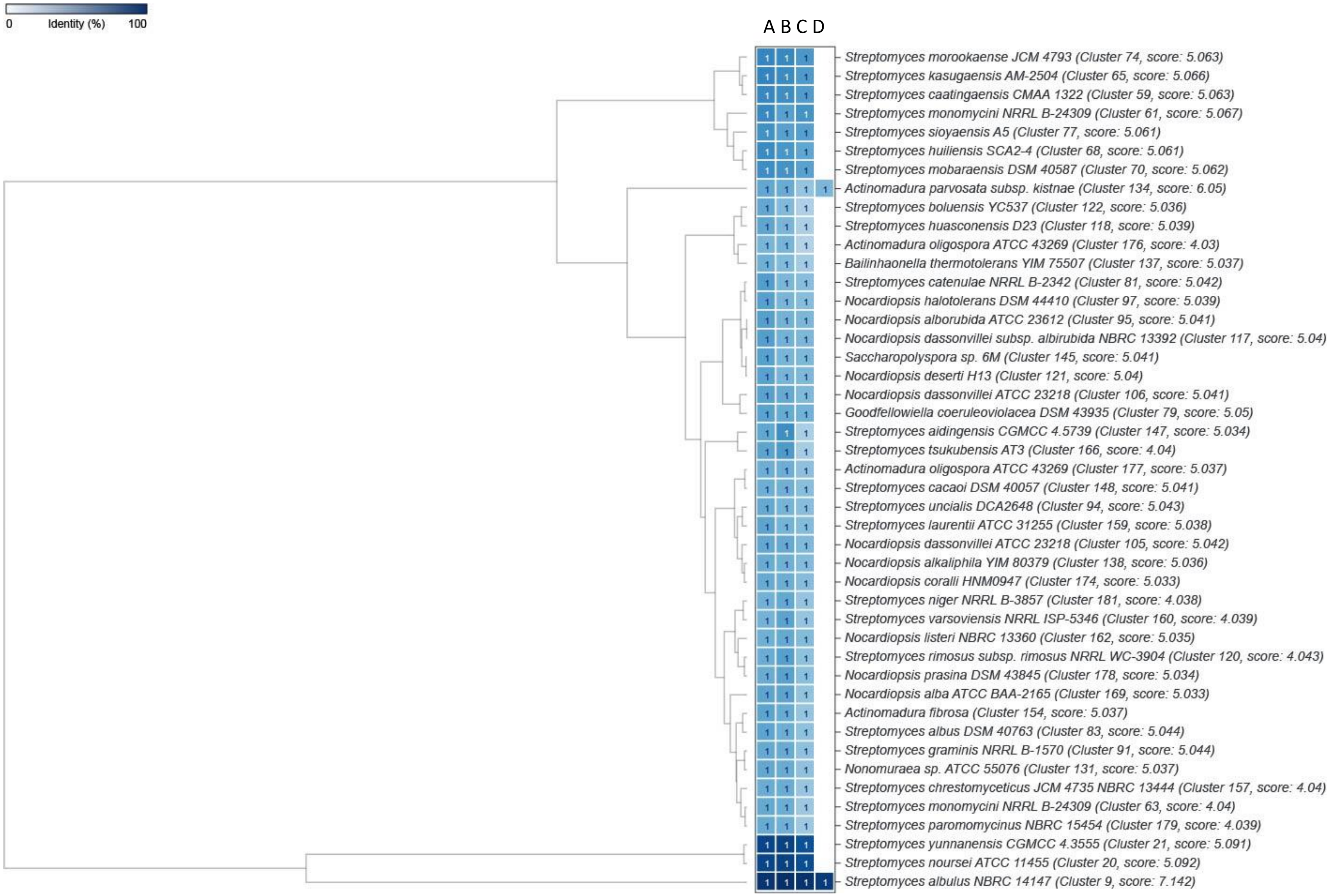


***Figures S5***. Distribution statistics of putative *cdp* gene analogues.

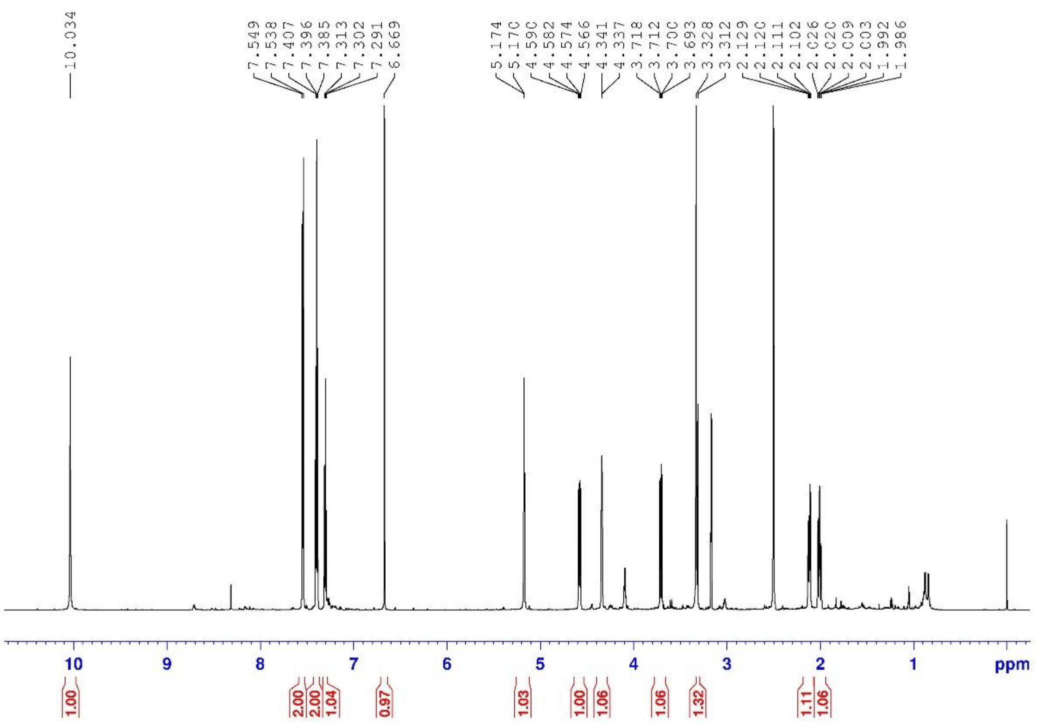


***Figure S6.*** $^{1}H$ NMR spectrum of **1** recorded in DMSO-$d_6$ (600 MHz)

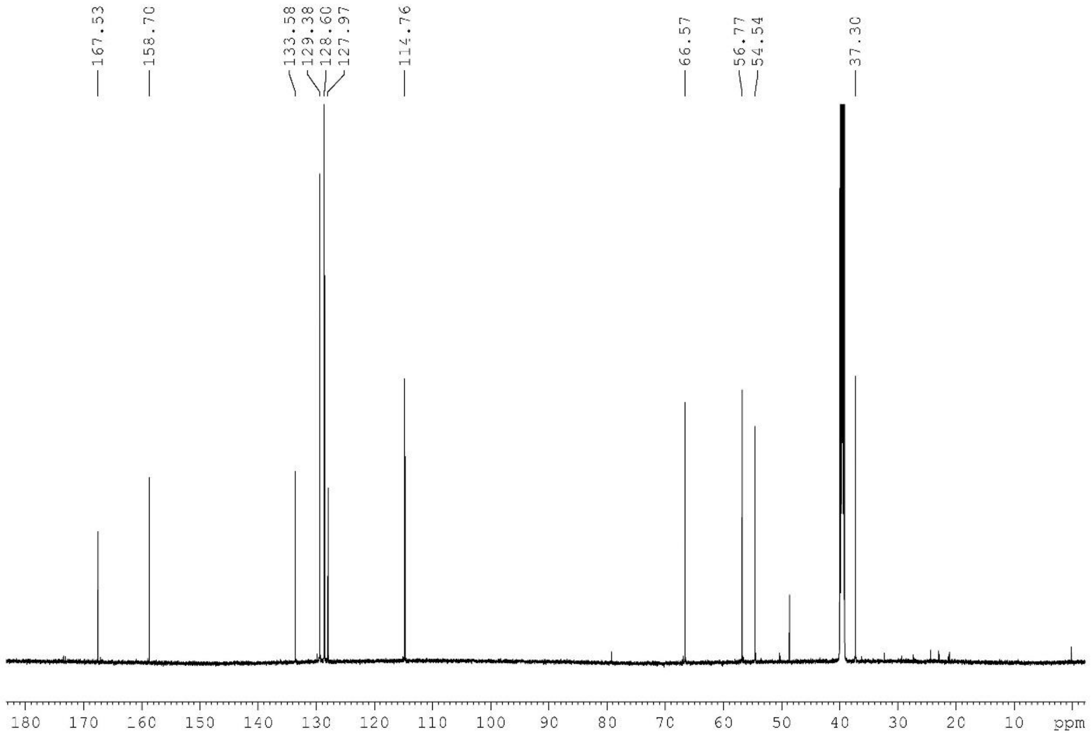


***Figure S7.*** $^{13}C$ NMR spectrum of **1** recorded in DMSO-$d_6$ (150 MHz)

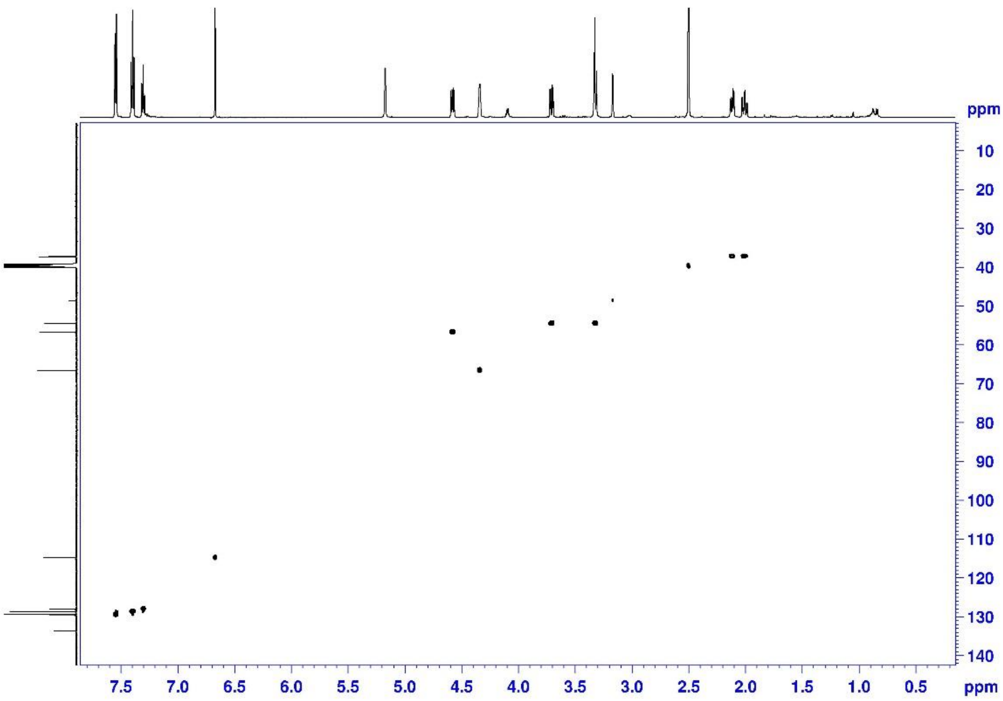


***Figure S8.*** HSQC spectrum of **1** recorded in DMSO-*d*$_6$ (600 MHz)

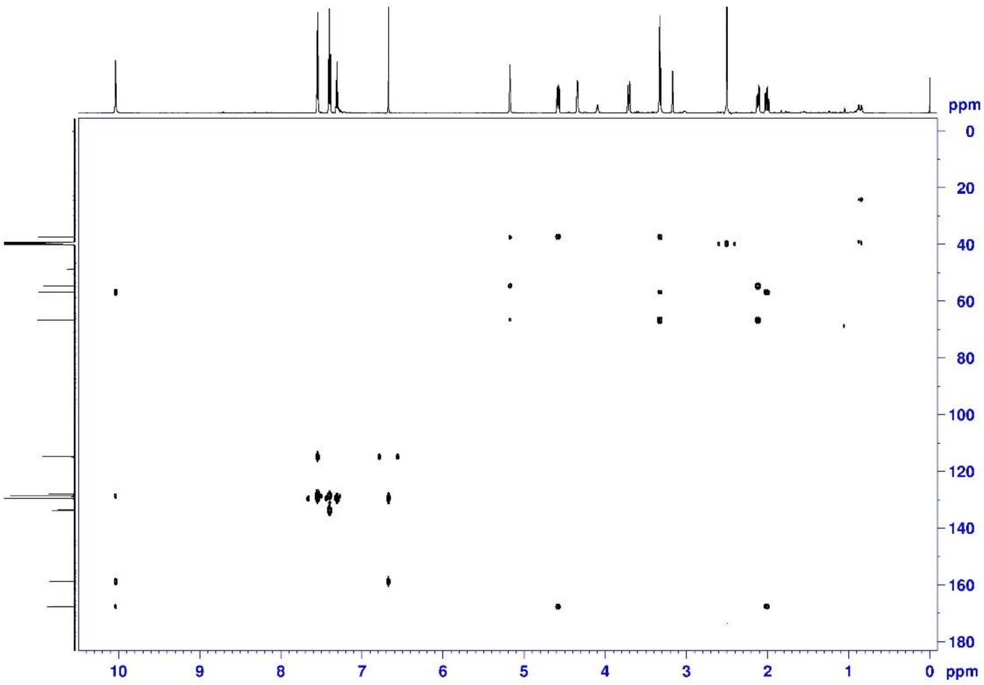


***Figure S9.*** HMBC spectrum of **1** recorded in DMSO-*d*$_6$ (600 MHz)

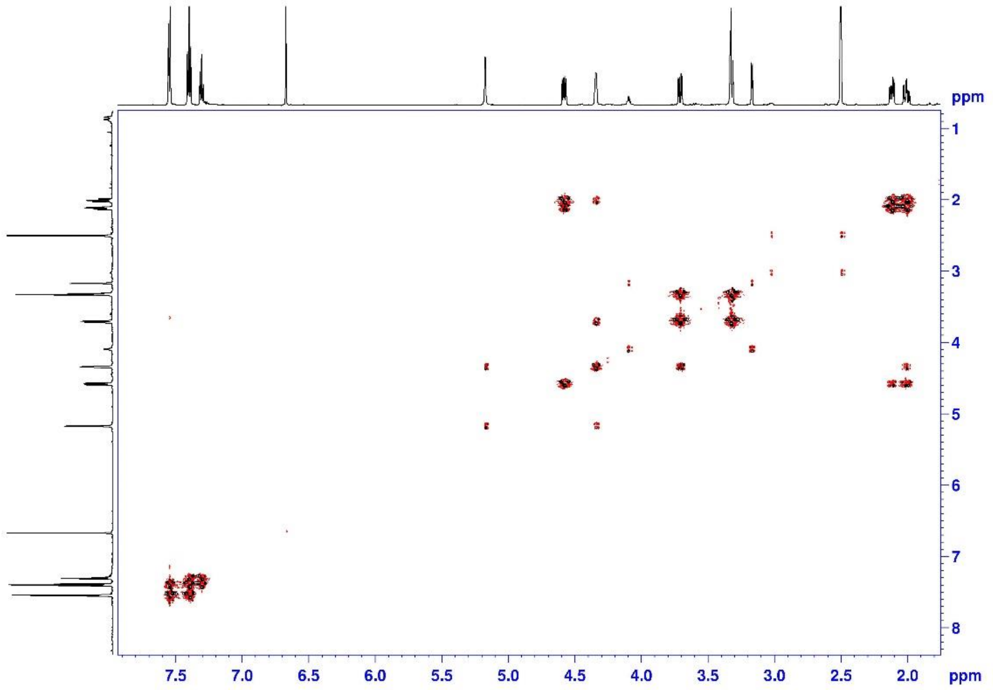


***Figure S10.*** $^{1}H$-$^{1}H$ COSY spectrum of **1** recorded in DMSO-$d_6$ (600 MHz)

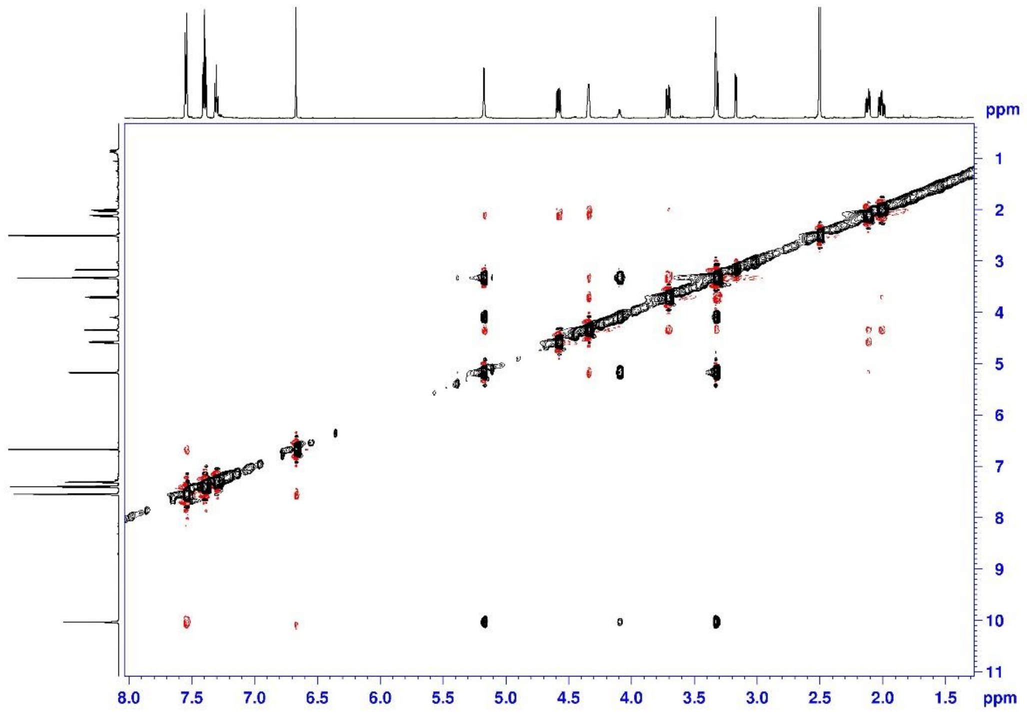


***Figure S11.*** $^{1}H$-$^{1}H$ NOSY spectrum of **1** recorded in DMSO-$d_6$ (600 MHz)

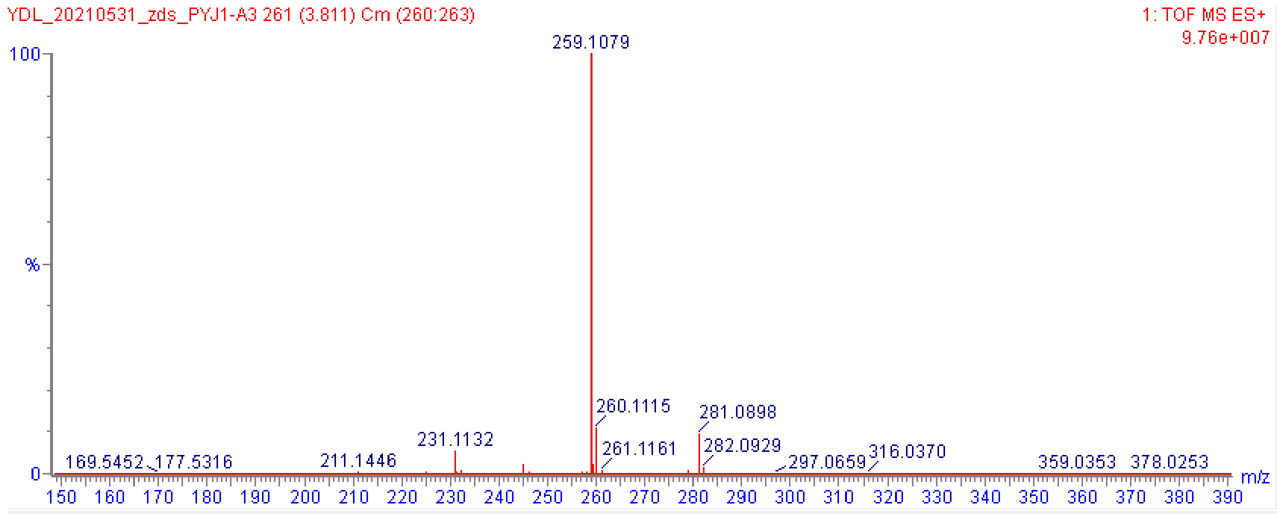


***Figure S12.*** HR-ESI-MS spectra of **1**

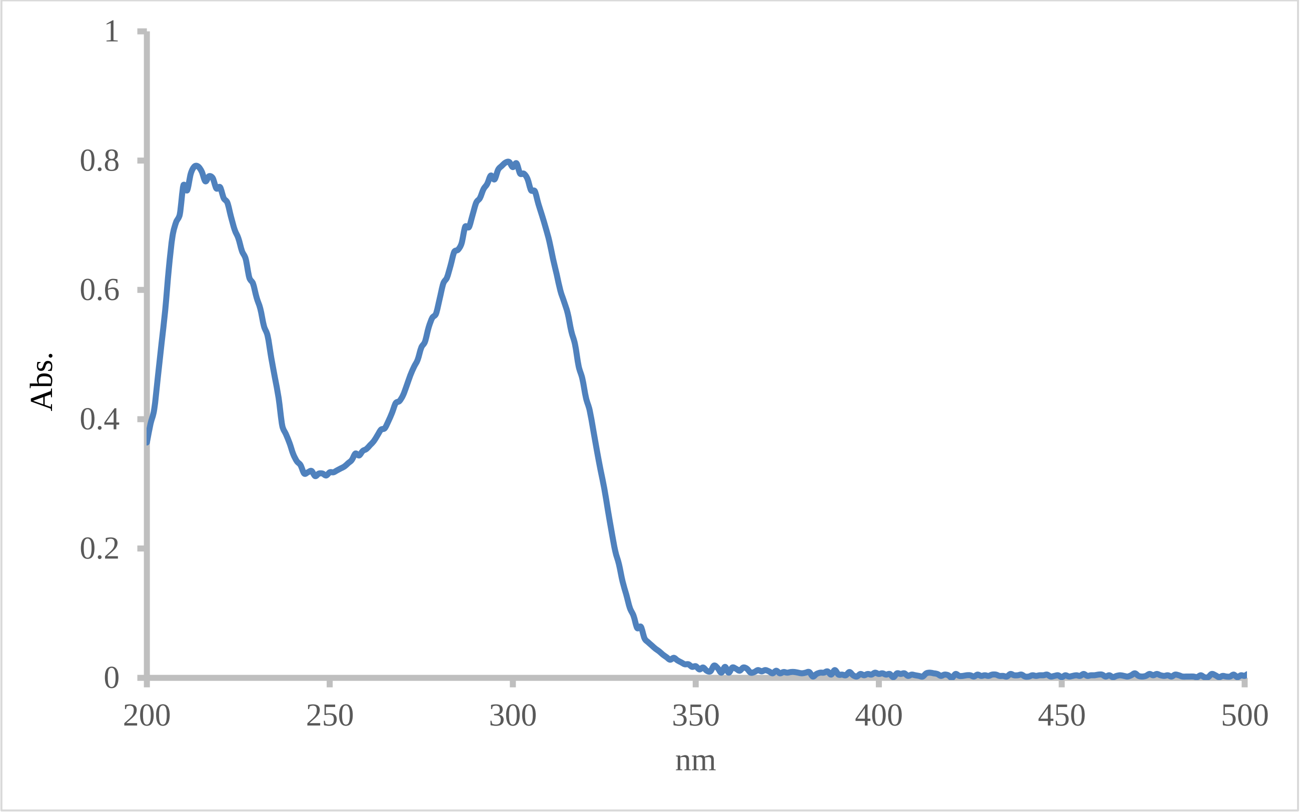


***Figure S13.*** UV spectrum of **1**

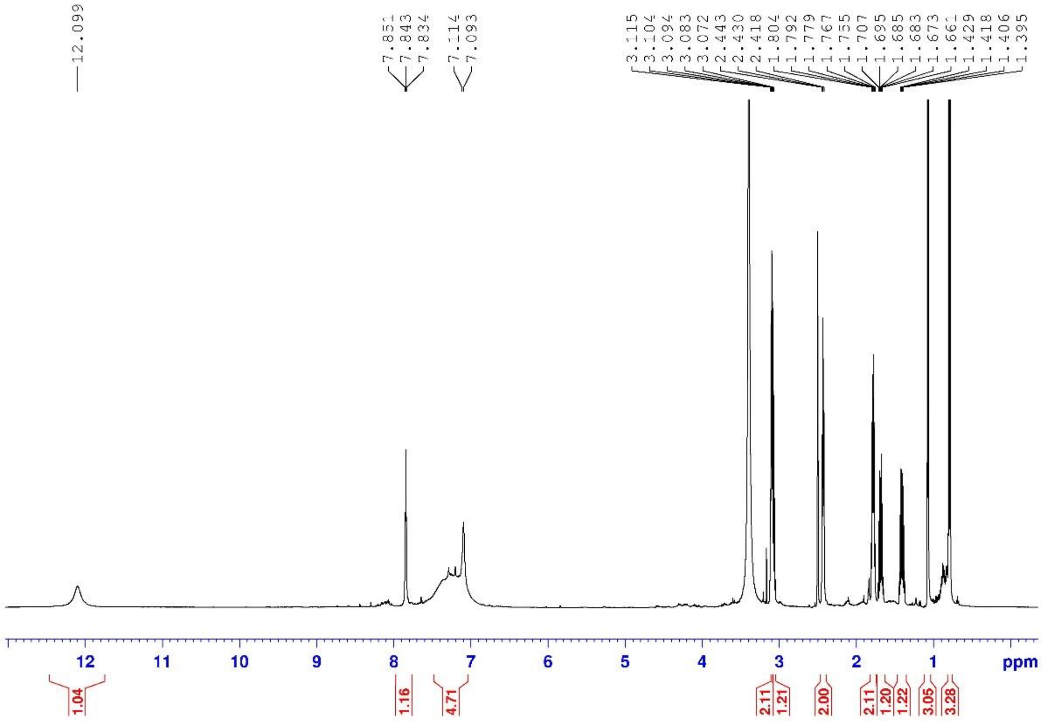


***Figure S14.*** $^{1}$H NMR spectrum of **2** recorded in DMSO-$d_6$ (600 MHz)

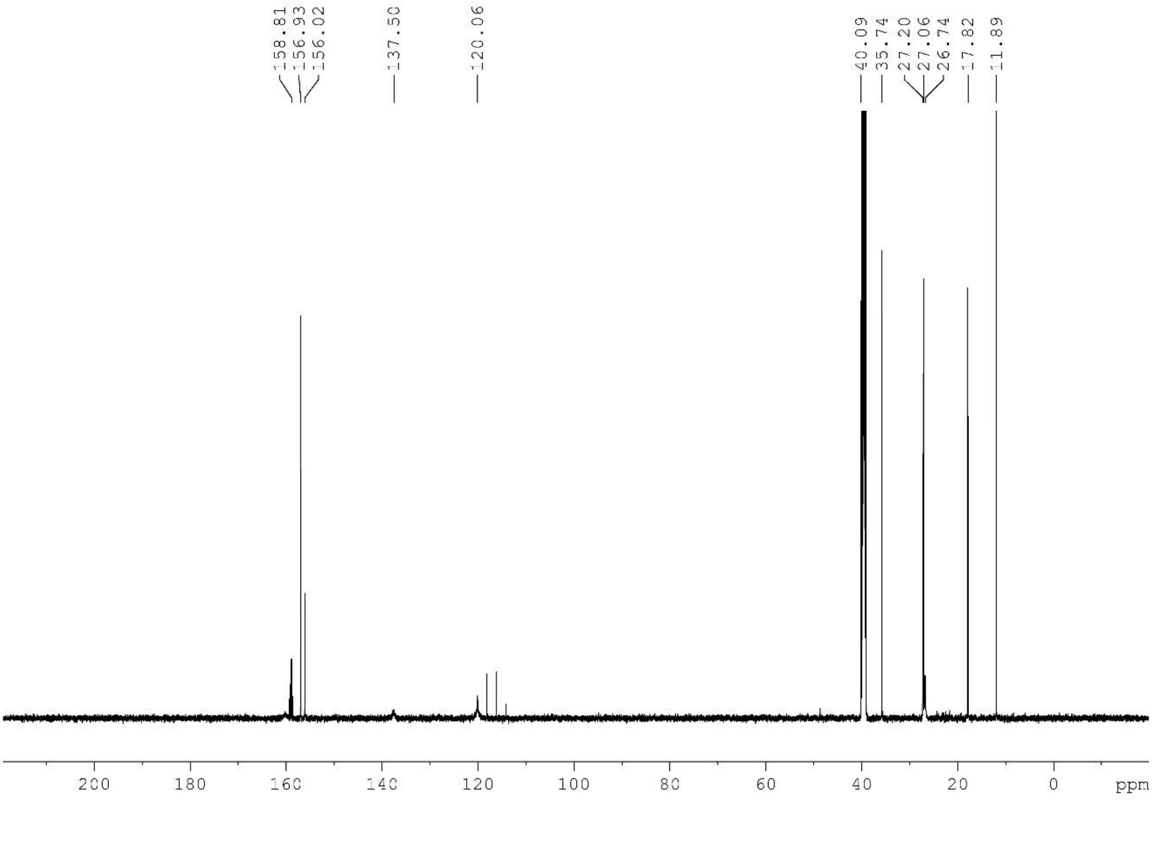


***Figure S15.*** $^{13}$C NMR spectrum of **2** recorded in DMSO-$d_6$ (150 MHz)

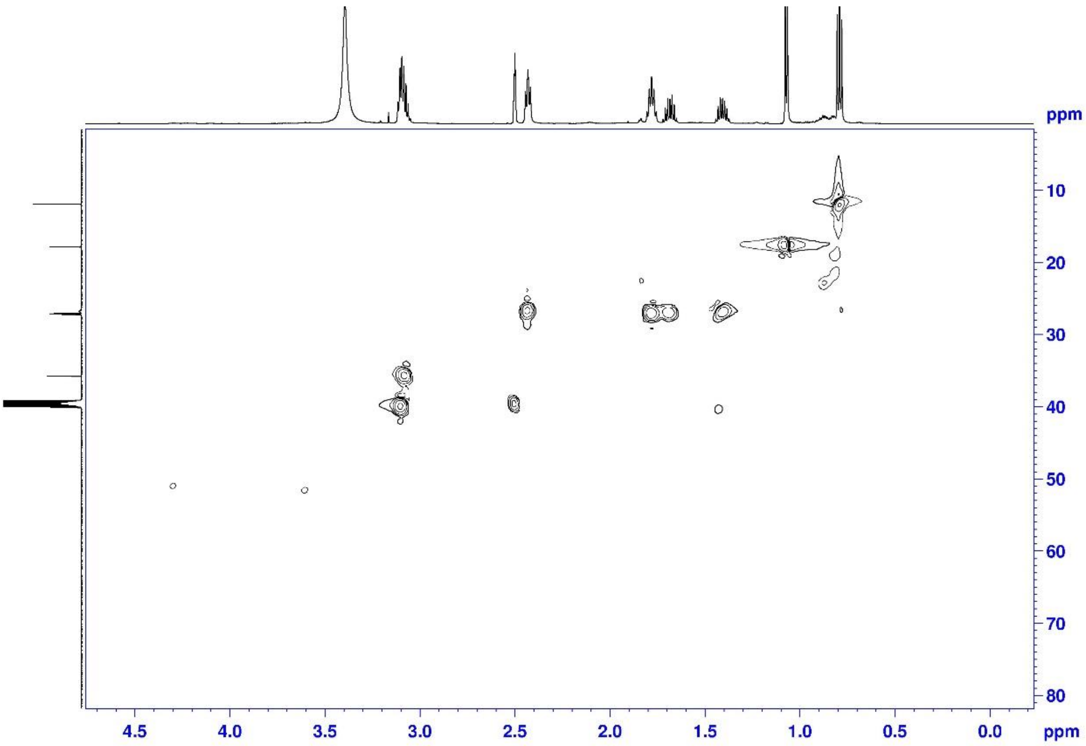


***Figure S16.*** HSQC spectrum of **2** recorded in DMSO-*d*$_6$ (600 MHz)

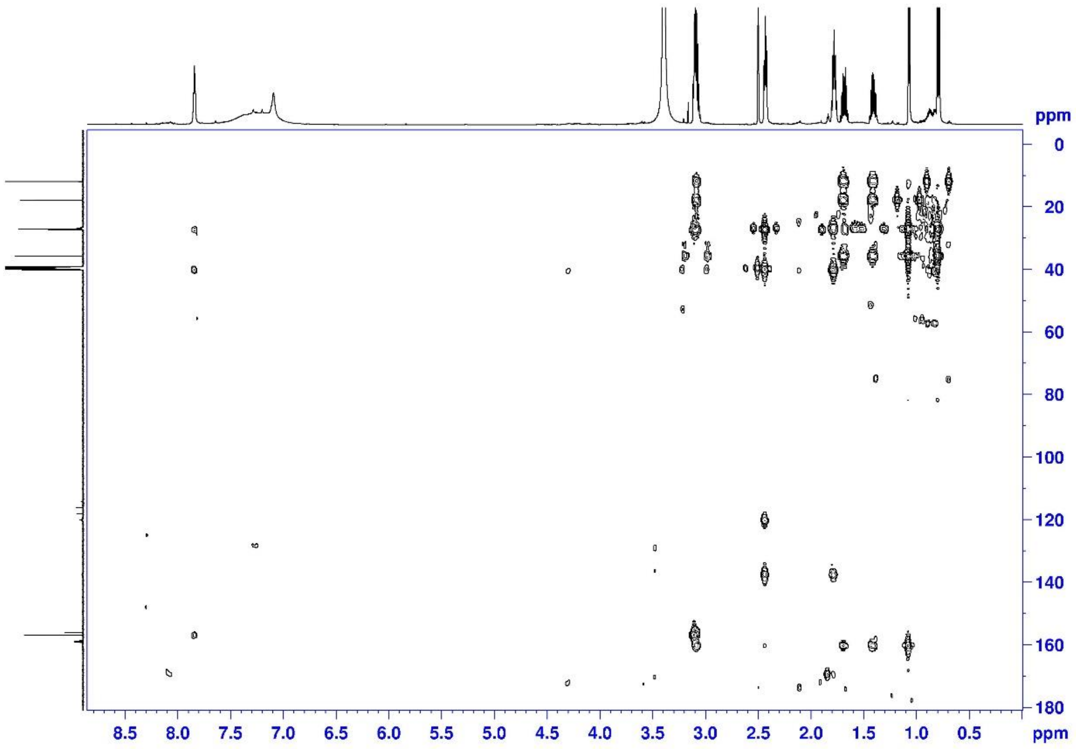


***Figure S17.*** HMBC spectrum of **2** recorded in DMSO-*d*$_6$ (600 MHz)

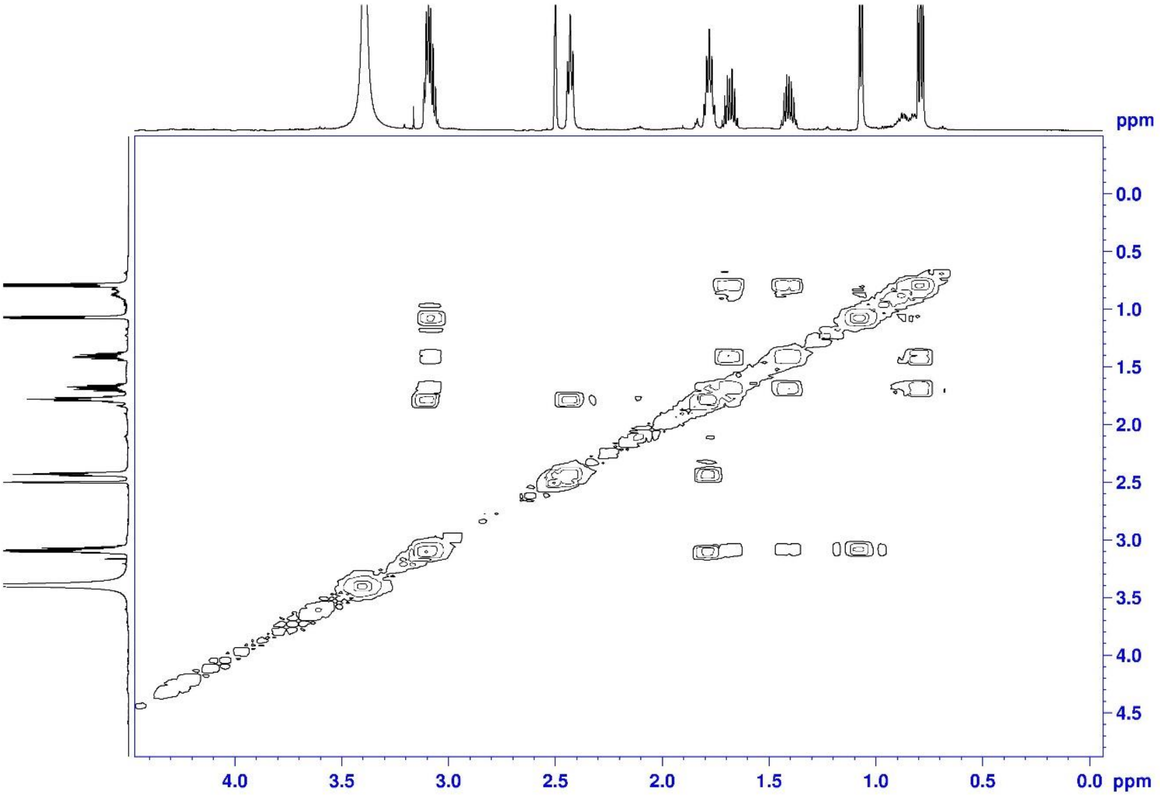


***Figure S18.*** $^1$H-$^1$H COSY spectrum of **2** recorded in DMSO-$d_6$ (600 MHz)

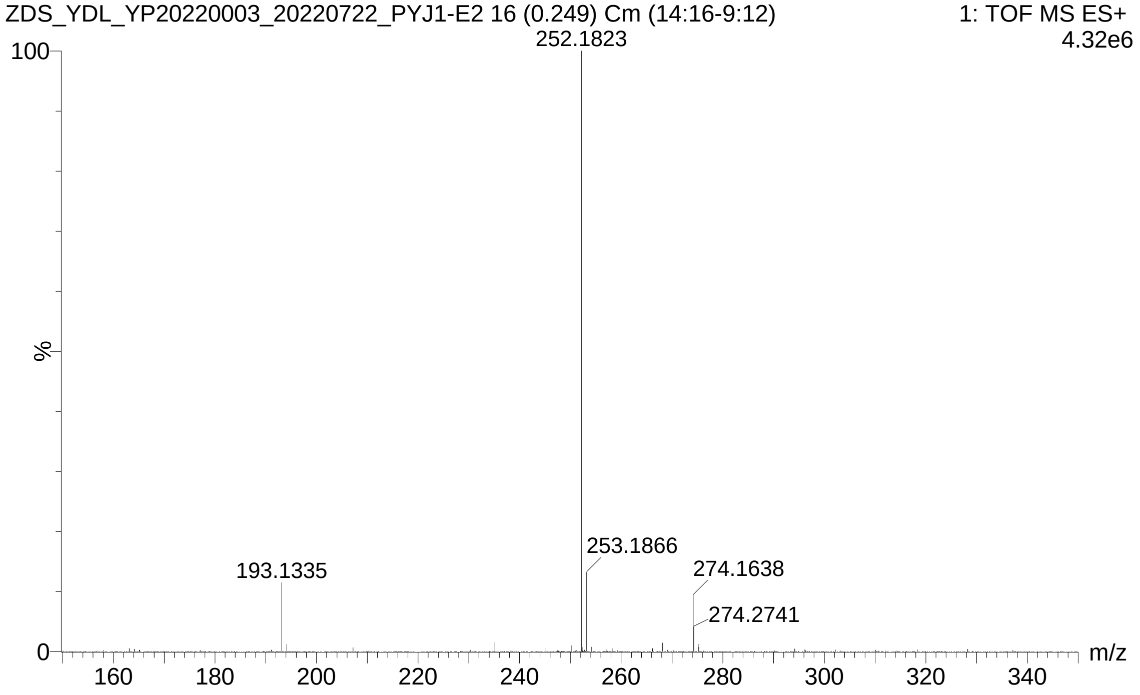


***Figure S19.*** HR-ESI-MS spectra of **2**

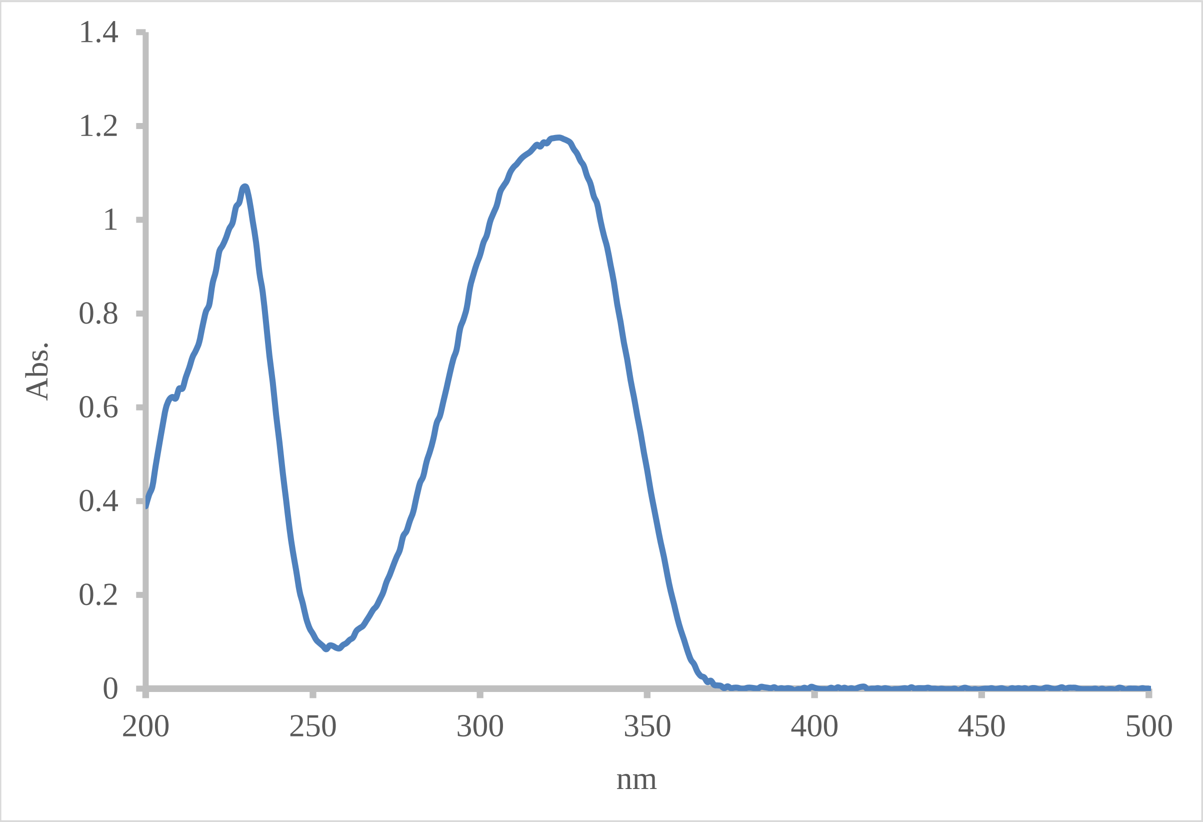


***Figure S20.*** UV spectrum of **2**

## References

18. Wang J.; Kong L.; Qiao Y.; Zhang D.; Deng Z.; You D. Genome mining guided discovery and identification of nystatin and toyocamycin in *Streptomyces hygrospinosus* var.beijingensis. *Acta Microbiol. Sin.* **2021**, 3076-3086. - 18 in main

38. Paget M. S.; Chamberlin L.; Atrih A.; Foster S. J.; Buttner M. J. Evidence that the extracytoplasmic function sigma factor sigmaE is required for normal cell wall structure in *Streptomyces coelicolor* A3(2). *J. Bacteriol.* **1999**, 181, 204-211.

39 He Y.; Wang Z.; Bai L.; Liang J.; Zhou X.; Deng Z. Two pHZ1358-derivative vectors for efficient gene knockout in *Streptomyces*. *J. Microbiol. Biotechnol.* **2010**, 20, 678-682.

40. Gust B.; Challis G. L.; Fowler K.; Kieser T.; Chater K. F. PCR-targeted *Streptomyces* gene replacement identifies a protein domain needed for biosynthesis of the sesquiterpene soil odor geosmin. *Proc. Natl. Acad. Sci. U. S. A.* **2003**, 100, 1541-1546.

41. Tan G. Y.; Deng K.; Liu X.; Tao H.; Chang Y.; Chen J.; Chen K.; Sheng Z.; Deng Z.; Liu T. Heterologous biosynthesis of spinosad: an Omics-guided large polyketide synthase gene cluster reconstitution in *Streptomyces*. *ACS Synth. Biol.* **2017**, 6, 995-1005.

42. Sun X.; Wang G.; Xiao H.; Jiang J.; Xiao D.; Xing B.; Li A.; Zhang Y.; Sun K.; Xu Y.; Guo L.; Yang D.; Ma M. Strepimidazoles A-G from the plant endophytic *Streptomyces* sp. PKU-EA00015 with inhibitory activities against a plant pathogenic fungus. *J. Nat. Prod.* **2020**, 83, 2246-2254.

43. Sun C.; Liu Z.; Zhu X.; Fan Z.; Huang X.; Wu Q.; Zheng X.; Qin X.; Zhang T.; Zhang H.; Ju J.; Ma J. Antitubercular ilamycins from marine-derived *Streptomyces atratus* SCSIO ZH16 ΔilaR. *J. Nat. Prod.* **2020**, 83, 1646-1657.

44. Perlatti B.; Lan N.; Earp C. E.; AghaAmiri S.; Vargas S. H.; Azhdarinia A.; Bills G. F.; Gloer J. B. Arenicolins: C-glycosylated depsides from *Penicillium arenicola. J. Nat. Prod.* **2020**, 83, 668-674.

45. Huo C.; Zheng Z.; Xu Y.; Ding Y.; Zheng H.; Mu Y.; Niu Y.; Gao J.; Lu X. Naphthacemycins from a *Streptomyces* sp. as protein-tyrosine phosphatase inhibitors. *J. Nat. Prod.* **2020**, 83, 1394-1399.

46. Zhang W.; Wang L.; Kong L.; Wang T.; Chu Y.; Deng Z.; You D. Unveiling the post-PKS

redox tailoring steps in biosynthesis of the type II polyketide antitumor antibiotic xantholipin. *Chem. Biol.* **2012**, 19, 422-432.

21. Lautru S.; Gondry M.; Genet R.; Pernodet J. L. The albonoursin gene cluster of *S. noursei* biosynthesis of diketopiperazine metabolites independent of nonribosomal peptide synthetases. *Chem. Biol.* **2002**, 9, 1355-1364.
